# POSTER SESSION 2: Abstracts 385–665

**DOI:** 10.1002/pmf2.12025

**Published:** 2025-01-05

THURSDAY

January 30, 2025

4:00 PM – 6:00 PM

